# The Tyrosine Kinase Btk Regulates the Macrophage Response to *Listeria monocytogenes* Infection

**DOI:** 10.1371/journal.pone.0060476

**Published:** 2013-03-27

**Authors:** Afitap Derya Köprülü, Renate Kastner, Sebastian Wienerroither, Caroline Lassnig, Eva Maria Putz, Olivia Majer, Benjamin Reutterer, Veronika Sexl, Karl Kuchler, Mathias Müller, Thomas Decker, Wilfried Ellmeier

**Affiliations:** 1 Division of Immunobiology, Institute of Immunology, Center for Pathophysiology, Infectiology and Immunology, Medical University of Vienna, Vienna, Austria; 2 Department of Microbiology and Immunobiology, Max F. Perutz Laboratories, University of Vienna, Vienna, Austria; 3 Institute of Animal Breeding and Genetics and Biomodels Austria, University of Veterinary Medicine, Vienna, Austria; 4 Institute of Pharmacology and Toxicology, University of Veterinary Medicine, Vienna, Austria; 5 Department of Molecular Genetics, Max F. Perutz Laboratories, Medical University of Vienna, Vienna, Austria; National Jewish Health and University of Colorado School of Medicine, United States of America

## Abstract

In this study we investigated the role of Bruton's tyrosine kinase (Btk) in the immune response to the Gram-positive intracellular bacterium *Listeria monocytogenes* (*Lm*). In response to *Lm* infection, Btk was activated in bone marrow-derived macrophages (BMMs) and *Btk* ^−/−^ BMMs showed enhanced TNF-α, IL-6 and IL-12p40 secretion, while type I interferons were produced at levels similar to wild-type (wt) BMMs. Although Btk-deficient BMMs displayed reduced phagocytosis of *E. coli* fragments, there was no difference between wt and *Btk* ^−/−^ BMMs in the uptake of *Lm* upon infection. Moreover, there was no difference in the response to heat-killed *Lm* between wt and *Btk* ^−/−^ BMMs, suggesting a role for Btk in signaling pathways that are induced by intracellular *Lm*. Finally, *Btk* ^−/−^ mice displayed enhanced resistance and an increased mean survival time upon *Lm* infection in comparison to wt mice. This correlated with elevated IFN-γ and IL-12p70 serum levels in *Btk* ^−/−^ mice at day 1 after infection. Taken together, our data suggest an important regulatory role for Btk in macrophages during *Lm* infection.

## Introduction

Bruton's tyrosine kinase (Btk) is one of the five members of the Tec kinase family. Mutations in the human *BTK* gene are the cause of X-linked agammaglobulinemia (XLA), a severe primary immunodeficiency characterized by defects in B lymphocyte development. A similar although weaker syndrome called x-linked immunodeficiency (*xid*) has been identified in mice, which is caused by a point mutation in the pleckstrin homology domain of Btk. A large number of biochemical and genetic studies have revealed important functions for Btk in B lymphocytes and provided comprehensive insight into how Btk regulates B cell development, differentiation and activation [1].

In recent years, the functions of Btk have also been extensively studied in the myeloid lineage such as in monocytes/macrophages [2] and in neutrophils [3]. Btk has been implicated in TLR signaling and interacts with TLR4, 6, 8 and 9 and also with MyD88, MyD88-adaptor-like protein (Mal) and IRAK-1 [4]. Upon TLR4 stimulation, macrophages from *xid* mice produce less TNF-α [5], nitric oxide (NO) [6] and reactive oxygen species (ROS) [7], and show impaired p65 phosphorylation [8]. *Btk* ^−*/*−^ macrophages produce also less heme oxygenase after TLR4 activation [9]. Blood monocytes from XLA patients have impaired phagocytic functions [10] and TNF-α production upon TLR2 or 4 stimulation [11], however other studies report similar [12] or enhanced cytokine production upon stimulation of TLR4, 7 and 8 in XLA monocytes [13], [14]. Notably, beside its functions as a positive regulator, Btk can also act as a negative regulator of TLR signaling. Btk phosphorylates Mal upon TLR2 and 4 stimulation, leading to the degradation of Mal and as a consequence to the downregulation of the immune response [15]. In addition, human Btk-deficient neutrophils displayed enhanced ROS production associated with more apoptosis upon TLR2 and 4 stimulation [3].

Although many studies addressed the role of Btk in monocytes/macrophages during TLR signaling using synthetic ligands, the role of Btk during infections with microbial pathogens is less clear. One widely accepted model to study the innate immune response of macrophages during infection is *Listeria monocytogenes* (*Lm*), a Gram-positive intracellular bacterium [16], [17]. In humans, *Lm* can cause listeriosis, which affects immune-compromised persons, pregnant women and neonates. After ingestion, *Lm* infects cells of the large intestine including macrophages. In case of a systemic infection, *Lm* disseminates to the liver and spleen and infects especially macrophages. The innate immune response to *Lm* involves the recruitment of phagocytic cells such as neutrophils, macrophages and natural killer (NK) cells. Key cytokines for the protection during early phases of infection with *Lm* are TNF-α, IL-12 and IFN-γ. After uptake by macrophages, *Lm* escapes from the phagosome and replicates in the cytoplasm of infected cells [16], [17]. Macrophages display distinct transcriptional responses to phagosomal and cytosolic *Lm* [18], including MyD88-dependent signaling originating from the cell surface and the phagosome. The concerted response drives expression of pro- and anti-inflammatory cytokines such as TNF-α, IL-12 and IL-10 and an IRF-3-dependent pathway from the cytosol, ultimately resulting in the expression of the type I interferon (IFN-I) IFN-β [19]. Pattern recognition receptors involved in *Lm* recognition are TLR2 [20] and intracellular receptors such as NOD-like receptors (NLR) or RIG-I-like receptors (RLR) [21]. In addition, the cytosolic nucleic acid sensor LRRFIP1 has been described to mediate IFN-β production induced by *Lm* [22]. In this study, we investigated the role of Btk in macrophages during *Lm* infection. To address the cellular response, Btk-deficient bone marrow-derived macrophages (BMMs) were analyzed at a biochemical and molecular level upon infection with *Lm*. Btk was activated in BMMs in response to *Lm* infection and *Btk* ^−/−^ BMMs showed enhanced levels of TNF-α, IL-6 and IL-12p40, while the production of IFN-I remained unaltered. Although Btk-deficient BMMs displayed reduced phagocytosis of *E. coli* fragments, there was no difference between wild-type (wt) and *Btk* ^−/−^ BMMs in the uptake of *Lm* upon infection. Moreover, there was no difference in the response to heat-killed *Lm* (hk*Lm*) or in response to the TLR2 ligand Pam_3_CSK_4_, between wt and *Btk* ^−/−^ BMMs, suggesting a role for Btk in signaling pathways that are induced by intracellular *Lm*. Finally, we observed that *Btk* ^−/−^ mice displayed enhanced resistance and thus an increased mean survival time upon *Lm* infection in comparison to wt mice. This correlated with elevated IFN-γ and IL-12p70 serum levels in *Btk* ^−/−^ mice at day 1 after infection. Taken together, our data indicate an important regulatory role for Btk in macrophages in response to *Lm* infection and provide novel and important insights as to how Btk may regulate macrophage function during infection.

## Materials and Methods

### Ethics statement

All animal experiments were evaluated by the ethics committees of the Medical University of Vienna and of the University of Veterinary Medicine Vienna and approved by the Federal Ministry for Science and Research, Vienna, Austria (GZ:BMWF-68.205/0233-II/10b/2009**).**

### Mice

WT (C57BL/6) or Btk-deficient mice [23] (Jackson Laboratory) in a C57BL/6 background (N8) were maintained in the animal facility of the Medical University of Vienna. Btk maps to the X chromosome and thus the genotype of Btk-deficient male mice is *Y/* ^−^; however, for simplicity we refer to Btk-deficient mice as *Btk* ^−/−^ mice throughout the article regardless of whether they were male or female. All mice used were 8–11 weeks old.

### Generation of BMMs

BMMs were generated as described before [24], [25]. In brief, wt and *Btk* ^−*/*−^ BM was isolated and after red blood cell lysis BM cells (10–15×10^6^) were plated on 10 cm bacteriological dishes (Sterilin). Cells were cultured in 10 ml culture medium per dish (DMEM (Sigma-Aldrich) supplemented with 10% FBS (PAA Laboratories), 100 units/ml penicillin, 100 µg/ml streptomycin (Sigma-Aldrich), 2 mM L-glutamine (Sigma-Aldrich), 10 mM HEPES (PAA Laboratories), 50 µM 2-Mercaptoethanol (Life Technologies) and 20% L929-cell-conditioned medium) at 37°C in a 5% CO_2_ atmosphere. Additional 5 ml culture medium was added on day 2 or 3 and subsequently the whole medium was replaced with 10 ml fresh medium every second day. The purity of the macrophage population was determined between day 8–10 by flow cytometry using anti-F4/80 (clone BM8, BioLegend) and anti-CD11b (clone M1/70, BD Biosciences) antibodies; BMMs were in general>95% of F4/80^+^CD11b^+^ cells. Samples were acquired on a FACS Calibur.

### *Lm* infection and activation of BMMs

*Lm* strains used in this study were LO28 [26] and EGD [27]. *Lm* strains were grown in brain heart infusion (BHI) broth at 37°C as previously described [27]. BMMs of day 8–10 cultures were harvested with citric saline buffer (10× stock: 1.35 M potassium chloride, 0.15 M sodium citrate) and seeded on tissue culture-treated (TC) multi-well plates (Nunc). Antibiotic-free medium was used for *Lm* infection. On the next day the cells were infected with LO28 at a multiplicity of infection (MOI) of 10. One hour after *Lm* infection, the culture medium was exchanged with medium containing gentamicin (50 µg/ml). After another hour, the medium was exchanged with medium containing 10 µg/ml gentamicin. For the activation with TLR2 ligands, BMMs were incubated with hk*Lm* (InvivoGen; 10^8^ cells/ml) or with Pam_3_CSK_4_ (InvivoGen; 1 µg/ml).

### Cytokine quantification

BMMs (2×10^5^ in 500 µl medium, 24 well plate) were infected as described above. Supernatants were collected 24 hours after infection. Cytokine levels in supernatants and in mouse serum were measured by ELISA (IL-12p70 and IFN-γ ELISAs were from eBioscience; TNF-α and IL-6 ELISAs were from BD Biosciences). IL-12p40 was measured using a cytometric bead array (CBA; BD Biosciences). Measurements were performed according to the manufacturers' protocols.

### IFN-I bio-assay and NO assay

IFN-I activity was measured using the indicator cell line L929-ISRE-luc, which contains a stable transfected luciferase construct driven by an interferon-sensitive regulatory element [28]. The indicator cells were plated on 96-well plates (2×10^4^ cells/100 µl/well) one day prior to the assay. The next day, supernatants of *Lm*-infected BMMs were added and the cells were incubated for 8–12 hours. Subsequently, the luciferase activity was determined using the ONE-Glo Luciferase Assay System (Promega). Recombinant IFN-β was used as a standard. NO levels in the supernatants were measured by a fluorometric assay kit (BioVision) according to the manufacturer's instructions.

### RNA extraction and semi-quantitative reverse transcription PCR (RT-PCR) analysis

BMMs (2×10^6^ in 2 ml medium, 6-well TC plate) were infected as described above. Cells were harvested with TRIZOL reagent (Invitrogen) and total RNA was isolated according to the manufacturer's instructions. RNA was reversely transcribed using SuperScriptII reverse transcriptase (Invitrogen) and the expression of Tec kinases and TLR2 was analyzed by RT-PCR. *Hprt* was used as an input control. The following primers were used: *Tlr2*-F: 5′-AAGAGGAAGCCCAAGAAAGC, *Tlr2*-R: 5′CGATGGAATCGATGATGTTG; *Bmx*-F*:* 5′-GCAGCCCTATGACTTATATGAT-3′ *Bmx*-R: 5′-CAGATAAACAGCACATAGACC; *Btk*-F*:* 5′-GTGTGTTCCACGCCACAGAG, *Btk*-R: 5′GACGCCATACAACTGCACCA; *Itk*-F: 5′-CCGCGACAAAGCTGAAAAAC, *Itk*-R: 5′-GAGAGTGGGGTTTGACCACG; *Rlk*-F: 5′-GCCAAACTGATGGGCAAAAC, *Rlk*-R: 5′-CTGGATCAACTCGGGGACTG; *Tec*-F: 5′-GCTGGAGAAAGAGCAACCAGA, *Tec*-R: 5′-ACACTGGTAACTCCCATCTGC; *Hprt*-F: 5′-ATTGTGGCCCTCTGTGTGCT; *Hprt*-R: 5′-TTGCGCTCATCTTAGGCTTTG.

### Phagocytosis assay, *Lm* uptake and phagosomal escape assays

BMMs (10^5^) were seeded on a 96-well TC plate in 100 µl culture medium, let adhere for one hour and incubated with 100 µg fluorescein-labeled *E. coli* particles for two hours. Phagocytosis was quantified using the Vybrant Phagocytosis Assay Kit (Molecular Probes) according to the manufacturer's instructions. Bacterial uptake and phagosomal escape assays were carried out according to a previously described protocol [27]. In brief, 5×10^5^ BMMs were seeded on coverslips in 6-well TC plates and on the next day infected with 10 µM CFSE-labeled LO28 at an MOI of 40. Three hours after infection, cells were fixed with 500 µl 4% paraformaldehyde for 10 min and permeabilized with 500 µl 0.1% Triton X-100 for 10 min. Subsequently, to detect the actin cytoskeleton, cells were incubated in 6.6 µM AlexaFluor 594 phalloidin (Molecular Probes) for 40 min at room temperature with two PBS washes between each step. Afterwards, cells were counterstained with DAPI (4′, 6-diamidino-2-phenylindole) at a final concentration of 250 ng/ml. To test the effect of exogenously added cytokines on bacterial escape, TNF-α (14,7 ng/ml), IL-6 (21 ng/ml) and IL-12 (1,5 ng/ml) were added to wt cultures during infection. The cytokines were purchased from Peprotech. Alternatively, *Lm* was visualized by using an α-*Lm* antibody (Abcam) and a secondary AlexaFluor 488 antibody (Molecular Probes). Samples were analyzed using a fluorescence microscope (Nikon) or a confocal microscope (Zeiss). The number of intracellular bacteria as well as the number of bacteria colocalizing with host F-actin were counted in 50 BMMs per assay. Phagosomal escape was expressed as the percentage of bacteria per cell that co-localize with F-actin.

### Preparation of whole and nuclear BMM lysates and immunoblot analysis

BMMs (2×10^6^ in 2 ml medium, 6-well TC plate) were infected as described above. For whole cell lysates, BMMs were lysed in Carin lysis buffer (20 mM Tris-HCl [pH 8.0], 138 mM NaCl, 10 mM EDTA, 100 mM NaF, 1% Nonidet P-40, 10% glycerol, 2 mM Na vanadate) supplemented with complete protease inhibitors (Roche). For nuclear protein, cells were fractionated using the NE-PER nuclear and cytoplasmic extraction reagents (Pierce Biotechnology) according to the manufacturer's instructions. Proteins were separated on 8 or 12% SDS-polyacrylamide gels and blotted onto PVDF membranes (Bio-Rad) using standard protocols. The following primary antibodies were used: HDAC1 (Millipore), Tubulin and Btk (Santa Cruz), IRF-3 (Zymed), phospho-Btk (Tyr223), phospho-IRF-3, ERK, phospho-ERK, JNK, phospho-JNK, p38 and phospho-p38 (Cell Signaling). Secondary antibodies were purchased from Jackson ImmunoResearch Laboratories. Signals were detected using ECL detection reagent (Amersham Biosciences) on the Fuji LAS-4000 imaging system. The band intensity was calculated using the MultiGauge software.

### *Lm* infection survival assays, serum preparation and CFU-assay

EGD (10^6^ CFU) was injected intraperitoneally into male mice. The survival of mice was monitored over a period of 14 days. For serum samples mice were anesthetized with ketamine-xylazine (100 mg ketamine/kg body weight and 4 mg xylazine/kg body weight; Ketasol and Xylasol, Graeub AG) and blood was collected retrobulbarly directly into serum tubes (Sarstedt Microvette 500). Serum was stored at −80°C until it was analyzed. To determine the bacterial load in spleen/liver, organs were isolated on day 3 or 5 post infection and homogenized in 2/4 ml endotoxin-free PBS (Sigma-Aldrich). Homogenates were diluted serially 1∶10 in PBS and plated in triplicates on Oxford-*Listeria*-selective agar plates (Merck Biosciences). After 2 days of incubation at 37°C, colonies were counted.

### Statistical analysis

All data are expressed as mean with SEM. Statistical analyses were carried out using the GraphPad Prism software. The P-values were calculated using either an unpaired Students t-test or a one sample t-test. Survival data were displayed as a Kaplan-Meier curve and analyzed using a log rank (Mantel-Cox) test. The P-values were defined as following: *, P<0.05; **, P<0.01; ***, P<0.001; n.s., not significant.

## Results

### Btk is activated in response to *Lm* infection

**Figure 1:**
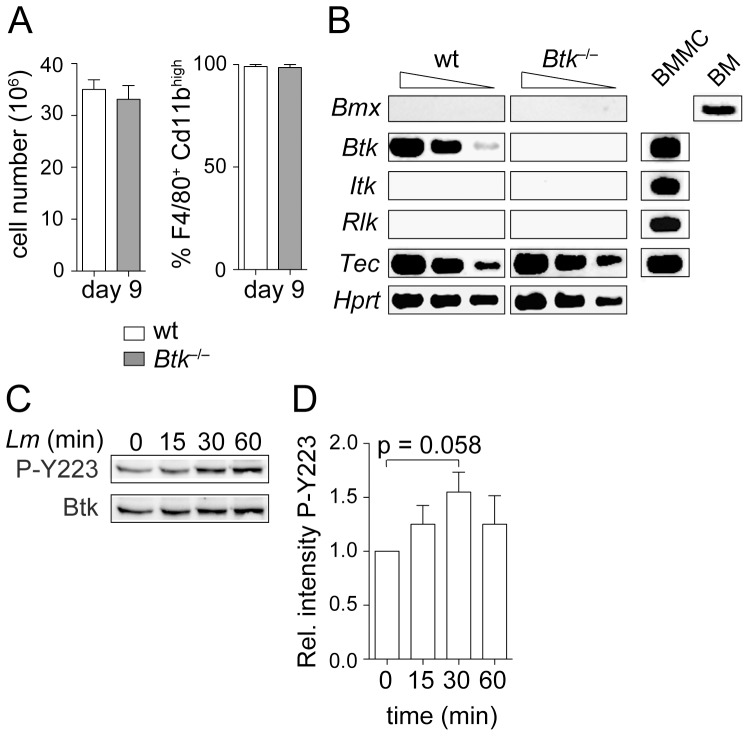
Btk is activated upon *Lm* infection. (A) Diagrams showing the total BMM cell number (left; n = 8) and the percentage of F4/80^+^CD11b^hi^ (right; n = 2) BMMs at day 9 of culture. Mean with SEM is shown. (B) RT-PCR analysis showing the expression of *Bmx*, *Btk*, *Itk*, *Rlk* and *Tec* in wt and *Btk* ^−*/*−^ BMMs. *Hprt* was used as a loading control. To confirm the functionality of the primers used for RT-PCR, the expression of *Btk*, *Itk*, *Rlk* and *Tec* in BM-derived mast cells (BMMC) and of *Bmx* in BM is shown. Data are representative of two independent experiments. (C) Immunoblot analysis showing the activation of Btk in wt BMMs. Cells were infected with *Lm* (LO28, MOI 10) for the indicated time periods and the autophosphorylation of Y223 was monitored in whole cell lysates. Total Btk levels serve as a loading control. Data are representative of four independent experiments. (D) Summary of the quantification of all immunoblots analyzed (n = 4). The relative intensity was calculated as the ratio of pY223-Btk/total Btk intensity for each time-point. Subsequently, the ratio in non-infected wt BMMs (time-point 0 min) was set as one and the relative levels for the other time points were calculated. The band intensity was evaluated using MultiGauge software. The P-value was calculated using an unpaired Student's t-test.

Macrophages play a crucial role in the early eradication of *Lm* [29]. To study the role of Btk in *Lm* infection in macrophages, we used BMMs generated from wt and *Btk* ^−/−^ mice. As previously reported [30], wt and *Btk* ^−/−^ BMM cultures displayed similar cell numbers (Fig. 1A, left) and similar differentiation frequency yielding F4/80^+^CD11b^hi^ cells (Fig. 1A). In addition to Btk, BMMs expressed also Tec, but none of the other Tec family members such as Bmx, Itk or Rlk ([30], Fig. 1B). However, there was no compensatory up-regulation of Tec in *Btk* ^−/−^ BMMs and there was also no induction of Bmx, Itk or Rlk expression in the absence of Btk (Fig. 1B).

To test whether Btk is part of the signaling cascade that is induced in macrophages upon *Lm* infection, wt and *Btk* ^−/−^ BMMs were infected with *Lm* for different time points. Subsequently, the phosphorylation status of the Btk autophosphorylation site Y223 was determined to assess Btk activity [31]. This revealed that Btk was activated approximately 30 min after *Lm* infection (Fig. 1C and 1D), indicating that Btk is part of the signaling network induced by *Lm*.

### Btk regulates cytokine production in BMMs in response to *Lm*

**Figure 2:**
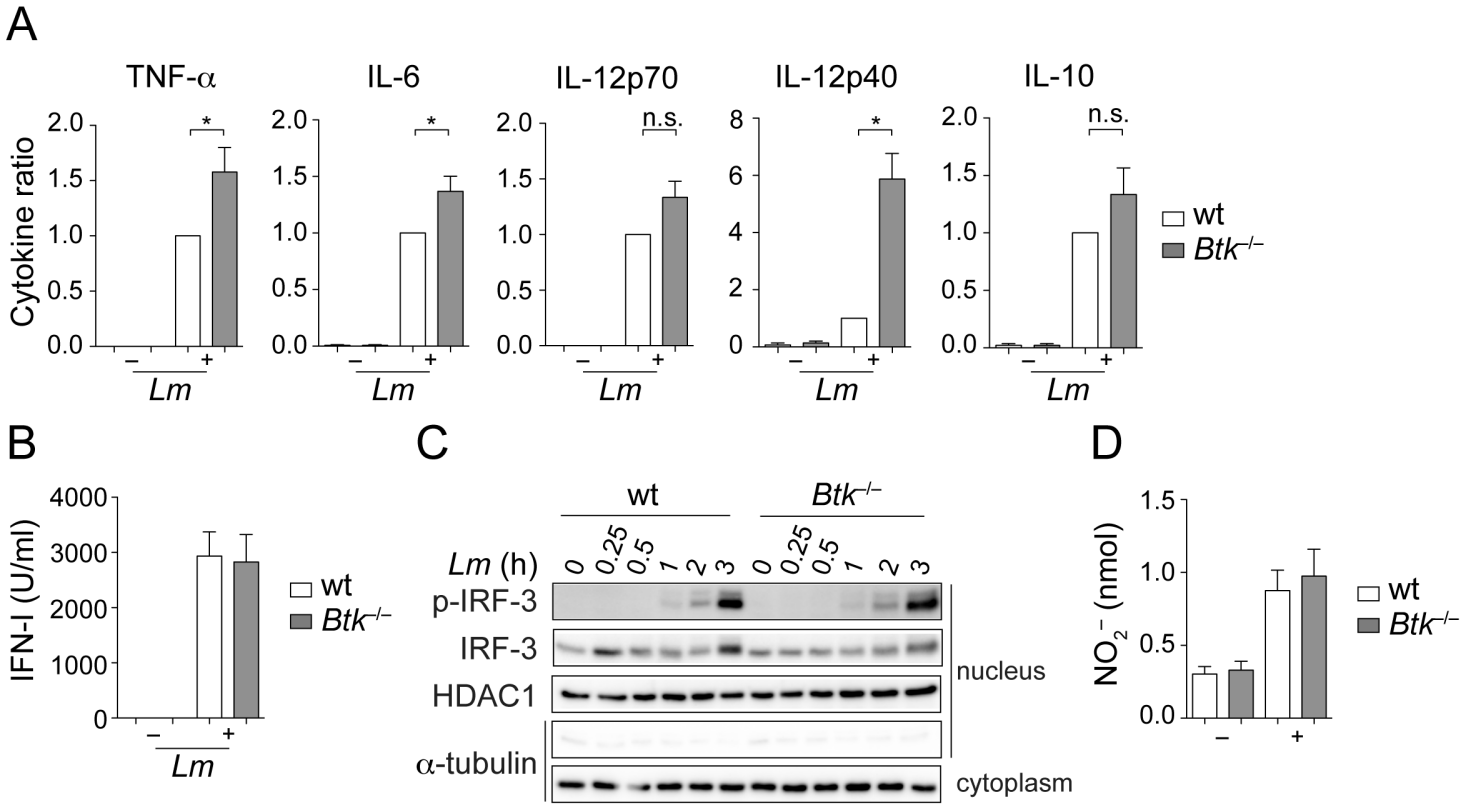
Btk regulates the cytokine response to *Lm*. (A) Wt and *Btk* ^−*/*−^ BMMs were infected (+) with *Lm* (LO28, MOI 10) or were left non-infected as a control (−). Twenty-four hours after infection, cytokine levels in the culture supernatants were determined with ELISA or with flow cytometric beads. Data show the summary of 9 (TNF-α, IL-10), 8 (IL-6) and 3 (IL-12p70, IL-12p40) experiments. The P-values were calculated using a one sample t-test. The cytokine levels in individual batches of wt BMMs were in the range of 4–9.8 ng/ml for TNF-α, 9.5–14.5 ng/ml for IL-6, 0.5–1.1 ng/ml for Il-12p70, 3–8 pg/ml for IL-12p40 and 0.3–1.5 ng/ml for IL-10. (B) Wt and *Btk* ^−*/*−^ BMMs were infected with *Lm* (LO28, MOI 10) (+) or were left non-infected (−). Twenty-four hours after infection, IFN-I levels in the supernatant were determined using the interferon-sensitive (ISRE) indicator cell line L929-ISRE (n = 3). (C) Nuclear translocation and phosphorylation kinetics of IRF-3 in nuclear extracts of *Lm*-infected (LO28, MOI 10) wt and *Btk* ^−*/*−^ BMMs. Tubulin indicates the purity of the nuclear extracts. HDAC1 is used as a loading control. (D) Wt and *Btk* ^−*/*−^ BMMs were infected with *Lm* (LO28, MOI 10) (+) or were left non-infected (−) and NO levels in the supernatants were assayed 24 hours later using a fluorometric assay (n = 6). Mean with SEM is shown. *, P≤0.05; n.s. not significant.

Next, we analyzed various effector functions of wt and *Btk* ^−/−^ BMMs in response to *Lm* infection *in vitro*. When compared to wt cells, *Btk* ^−/−^ BMMs showed increased production of the inflammatory cytokines TNF-α, IL-6 and IL-12p40 (Fig. 2A). Moreover, there was a tendency for enhanced IL-12p70 and IL-10 production, although it did not reach statistical significance (Fig. 2A). In contrast, IFN-I production was not altered in the absence of Btk (Fig. 2B). This correlated with a similar nuclear localization and phosphorylation status of IRF-3 upon *Lm* infection (Fig. 2C). We also determined the production of NO, another effector molecule produced by macrophages playing a central role in pathogen killing [32]. However, NO production upon *Lm* infection was similar in wt and *Btk* ^−/−^ BMMs (Fig. 2D). Together, these data demonstrate that *Lm* induces enhanced cytokine production, suggesting that Btk negatively regulates cytokine response in BMMs.

**Figure 3:**
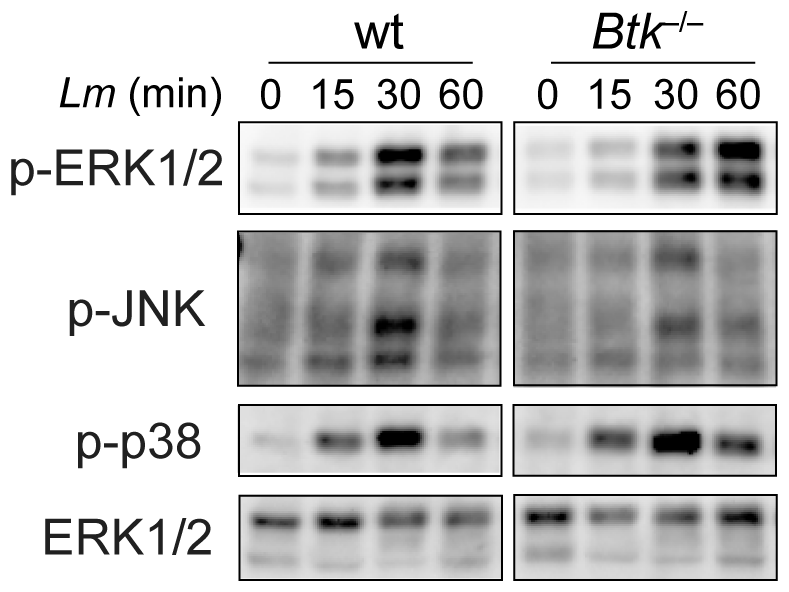
Immunoblot analysis of MAP kinase signaling. Immunoblot analysis showing the activation of Erk1/2, p38 and JNK1/2 in Wt and *Btk* ^−*/*−^ BMMs. The cells were infected with *Lm* (LO28, MOI 10) for the indicated time period. Total ERK1/2 protein levels served as a loading control. Data are representative of three independent experiments. Sustained activation of Erk1/2 has not been observed in the other two batches.

To further study the response of Btk-deficient BMMs, we determined the activation of well-known MAP kinase pathways. Wt and *Btk* ^−*/*−^ BMMs were infected with *Lm* and phosphorylation of ERK1/2, JNK and p38 was determined at various time-points after infection. This revealed a similar activation pattern of ERK1/2, JNK and p38 both in wt and Btk-deficient BMMs (Fig. 3), suggesting that a lack of Btk does not lead to a general signaling defect.

### The uptake of *Lm* is normal in Btk^−/−^ BMMs but phagosomal escape is impaired

**Figure 4:**
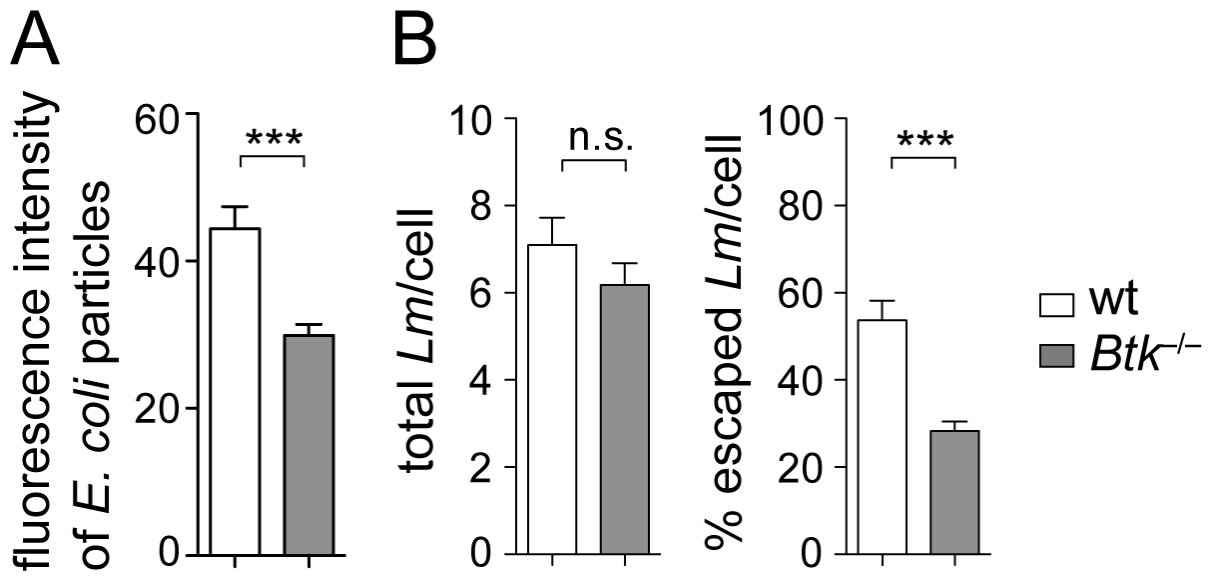
Analysis of the phagocytic function of *Btk* ^−*/*−^ BMMs and uptake of *Lm.* (A) Wt and *Btk* ^−*/*−^ BMMs were incubated with fluorescein-labeled *E. coli* particles for two hours and the fluorescence of phagocytosed particles was measured. Diagram indicates the fluorescence intensity by phagocytosed *E. coli* particles. Data show the summary of three independent experiments with a total of four independent cell batches. (B) Wt and *Btk* ^−*/*−^ BMMs were infected with CFSE-labeled *Lm* (LO28, MOI 40) and three hours later the cells were fixed, permeabilized and cellular actin was stained with Phalloidin-Alexa. The total number of intracellular bacteria and the number of cytoplasmic bacteria co-localizing with host actin were determined by fluorescence microscopy as described in material and methods. The diagram on the left indicates the mean number of *Lm* per cell. The right diagram displays the percentage of *Lm* that escaped to the cytoplasm. For each experiment, the number of *Lm* in 50 infected wt or *Btk* ^−*/*−^ BMMs was counted. Data are representative of two independent experiments. (A and B) Mean with SEM is shown. The P-values were calculated using an unpaired Student's t-test. ***, P≤0.001; n.s. not significant.

Upon uptake into the host cell, *Lm* becomes encapsulated in the maturing phagosome, from which it can escape into the cytosol for subsequent proliferation and spreading to neighboring cells [29]. Notably, Btk has been implicated in the regulation of phagocytosis in the macrophage cell line RAW 264.7 [33]. In agreement with these studies, we also observed that Btk-deficient BMMs displayed a reduced uptake of fluorescein-labeled *E. coli* fragments (Fig. 4A). To test whether the different cytokine response of Btk-deficient BMMs is due to a different uptake of *Lm*, we determined the numbers of bacteria that were internalized by wt and *Btk* ^−/−^ BMMs. The uptake of CFSE-labeled *Lm* was identical in wt and *Btk* ^−/−^ BMMs (Fig. 4B). Next, we quantified the escape rate of *Lm* from phagosomes by counting the numbers of *Lm* residing in the cytoplasm of infected macrophages. This revealed that *Btk* ^−/−^ BMMs show a reduced *Lm* escape into the cytosol 3 h after addition of *Lm* when compared to wt BMMs (Fig. 4C). Similar results were obtained using an α*-Listeria monocytogenes* antibody for the detection of *Lm* (Fig. S1). Taken together, these data indicate that alterations in the cytokine response in the absence of Btk were not due to differences in the uptake rate of *Lm.*

### Btk^−/−^ BMMs exhibit a normal cytokine response to hkLm and Pam_3_CSK_4_

**Figure 5:**
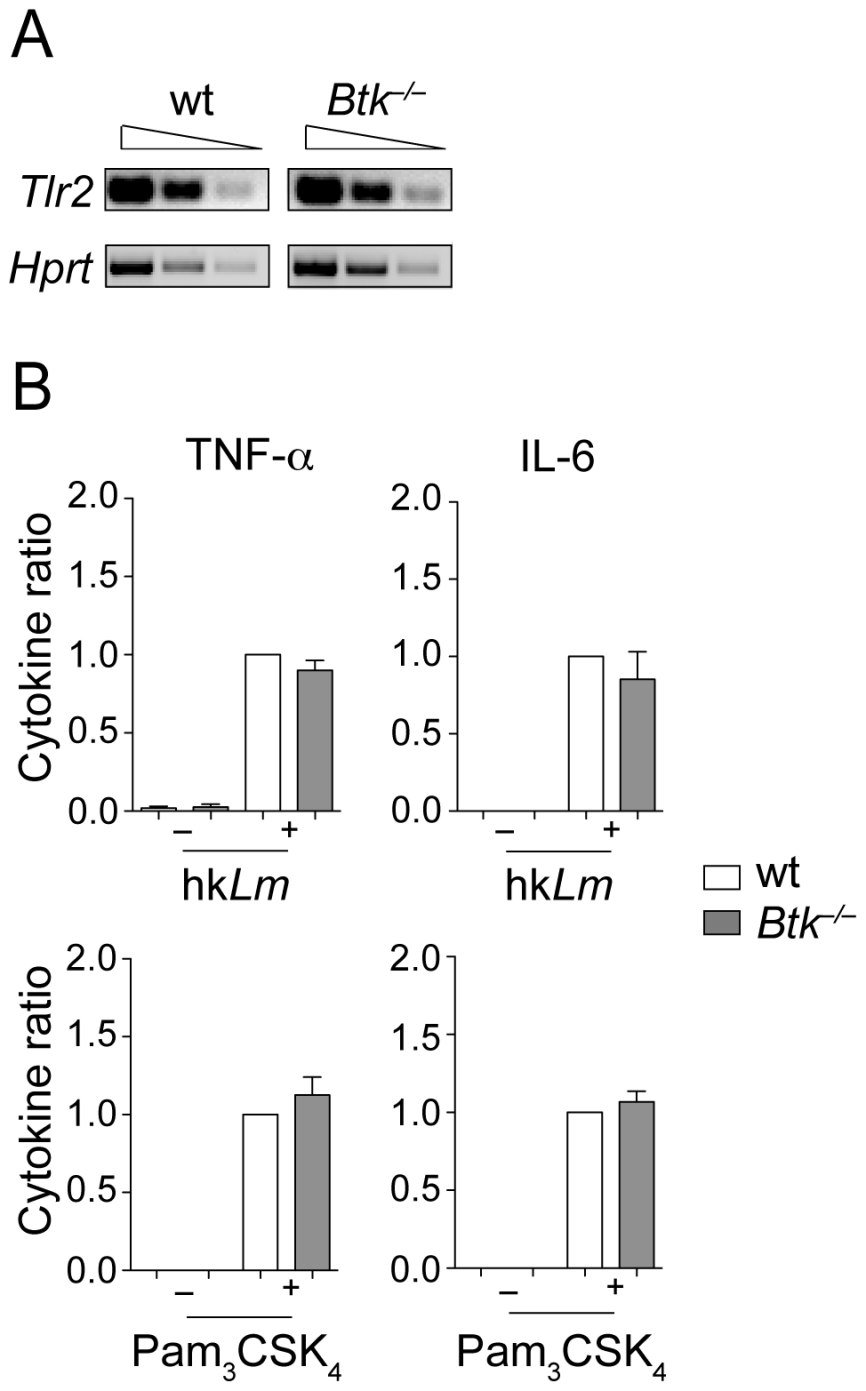
Normal cytokine production in response to hk*Lm* and Pam_3_CSK_4_. (A) Semi-quantivative RT-PCR analysis showing the expression of *Tlr2* in wt and *Btk* ^−*/*−^ BMMs. *Hprt* was used as an input control. Data are representative of two independent experiments. (B) Wt and *Btk* ^−*/*−^ BMMs were stimulated (+) with hk*Lm* (10^8^ cells/ml) or with Pam_3_CSK_4_ (1 µg/ml), or were left non-treated as a control (−) for 24 hours. Afterwards, the cytokine levels in the supernatants were determined by ELISA. Data show the summary of 4–13 independent experiments (hk*Lm*: TNF-α, n = 4, IL-6, n = 5; Pam_3_CSK_4_: TNF-α, n = 8, IL-6, n = 9). For each experiment, wt cytokine levels were set as 1 and the relative levels in supernatants from *Btk* ^−*/*−^ BMMs were calculated. Mean with SEM is shown. The cytokine levels in individual batches of wt BMMs for hk*Lm* were in the range of 2.3–9.6 ng/ml for TNF-α and 0.9–13.4 ng/ml for IL-6. For Pam_3_CSK_4_the range was 0.6–19.1 ng/ml for TNF-α and 2.3–39.5 ng/ml for IL-6.

To further characterize the response of *Btk* ^−/−^ BMMs to *Lm*, wt and *Btk* ^−/−^ BMMs were stimulated with hk*Lm* or with Pam_3_CSK_4_, both of which signal via TLR2 [34], [35]. The expression of TLR2 was similar in wt and *Btk* ^−/−^ BMMs (Fig. 5A). Thus potential functional differences in the response to *Lm* cannot be attributed to different TLR2 expression levels. In contrast to the experimental setup where BMMs were infected with *Lm*, there was no difference in the production of TNF-α and IL-6 upon stimulation with hk*Lm* (Fig. 5B). Moreover, the cytokine production in response to Pam_3_CSK_4_ was also unaffected in the absence of Btk (Fig. 5B). Together, these data indicate that *Btk* ^−/−^ BMMs display an altered cytokine production in response to *Lm* infection, but not in response to ligands that signal through TLR2.

### Btk^−/−^ mice display reduced susceptibility to *Lm* infection

**Figure 6:**
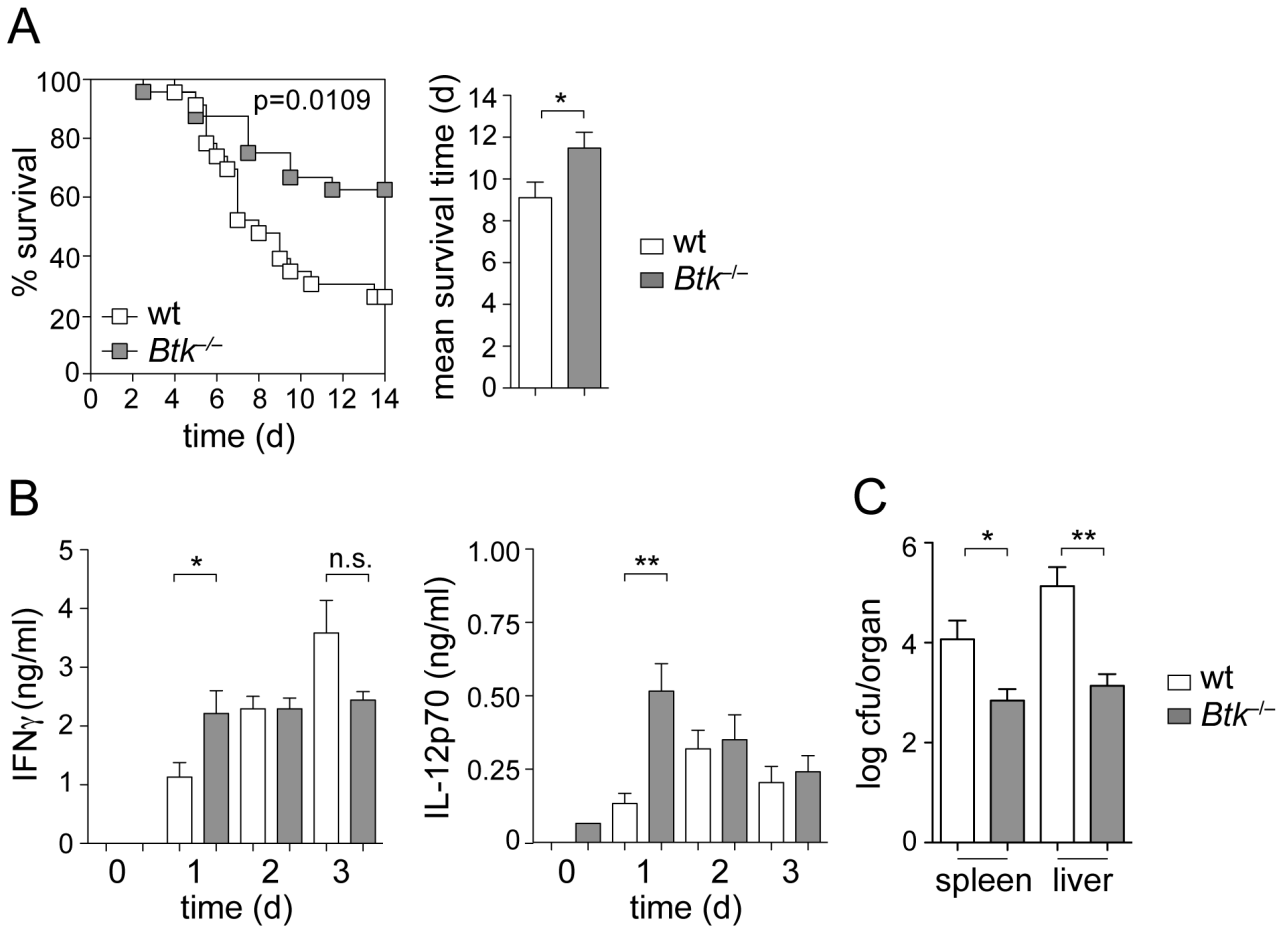
Btk-deficient mice show reduced susceptibility to *Lm* infection. (A) Left panel: Kaplan-Meier plot showing the survival of wt and *Btk* ^−*/*−^ mice after *Lm* (EGD, 10^6^ CFU) infection over a period of 14 days. Right panel: Diagram indicates the mean survival time of wt and *Btk* ^−*/*−^ mice after *Lm* infection. Data show the summary of two independent experiments with a total of 23 wt and 24 *Btk* ^−*/*−^ mice. (B) Wt and *Btk* ^−*/*−^ mice were infected with *Lm* (EGD, 10^6^ CFU) and serum IFN-γ (left) and IL-12p70 (right) levels were measured on day 1, 2 and 3. n = 5 (day 1), 6 (day 2), 5 (day 3) for IFN-γ and 5 (day 1), 5 (day 2), 5 (day 3) for IL-12p70. (C) Wt and *Btk* ^−*/*−^ mice were infected with *Lm* (EGD, 10^6^ CFU) and CFU-spleen and CFU-liver were measured on day 5. n = 5 (wt) and 8 (Btk-null). (A–C) Mean with SEM is shown. Survival data were analyzed using a log rank (Mantel-Cox) test, while the other P-values were calculated using an unpaired Student's t-test. *, P≤0.05; **, P≤0.01; n.s. not significant.

To determine whether loss of Btk activity affects the susceptibility of mice to *Lm* infection, wt and *Btk* ^−/−^ mice were infected with *Lm* by intraperitoneal injection, and the survival of mice was monitored over a period of 2 weeks. While approx. 25% of wt mice survived the infection at day 14, more than 60% of *Btk* ^−/−^ mice survived *Lm* infection at that time (Fig. 6A). As a consequence, the mean survival time of *Btk* ^−/−^ mice was enhanced in comparison to wt mice (Fig. 6A). Because IFN-γ plays a critical role in clearing infections during the early response to *Lm* [36], and since it is known to be induced by IL-12 [37], we determined serum levels of IFN-γ and IL-12. Correlating with the enhanced survival of *Btk* ^−/−^ mice, both IFN-γ and IL-12 (Fig. 6B) levels were increased in the serum of *Btk* ^−/−^ mice at day 1 after infection. Moreover, the bacterial load in spleen and liver was reduced at day 5 in Btk-deficient mice (Fig. 6C). These data indicate that the altered cytokine response of Btk-deficient macrophages to *Lm in vitro* correlates with an enhanced resistance of *Btk* ^−/−^ mice upon *Lm* infection. Taken together, our data suggest a role for Btk signaling in modulating virulence of the bacterial pathogen *Lm.*

## Discussion

In this study, we demonstrate a function for Btk in the innate immune response to *Lm* infections. We show that Btk-deficient macrophages display elevated levels of the pro-inflammatory cytokines TNF-α, IL-6 and IL-12, suggesting that Btk negatively regulates the extent of the inflammatory response in macrophages. Moreover, Btk-deficient mice are more resistant to *Lm* infection when compared to wt mice and the increased survival correlates with increased IL-12 and IFN-γ levels in the serum and reduced bacterial loads in spleens and livers of infected mice. Taken together, our data indicate an important and as yet unrecognized regulatory role for Btk in macrophages in response to *Lm* infection. The work provides novel insights into how Btk may regulate macrophage function during infection.

At the cellular level, the innate immune response to *Lm* infection is controlled by various cell types including phagocytes such as neutrophils and macrophages, and NK cells [16]. In this study we focused our analysis on macrophage function in the absence of Btk. Macrophages are known to release pro-inflammatory cytokines upon uptake of *Lm*, which is important for the induction of the subsequent immune response [16]. TNF-α and IL-12 secreted by macrophages upon Lm infection stimulate IFN-γ production by NK cells which in turn activates bactericidal activities of macrophages [16], however pro-inflammatory cytokines also induce dendritic cells (DC) [38] and neutrophils [28] to produce IFN-γ in response to Lm infection. Therefore, our finding that pro-inflammatory cytokine production was enhanced in Btk-null macrophages might explain the enhanced serum IFN-γ levels at day 1 following Lm infection and hence the increased resistance of *Btk* ^−*/*−^ mice upon Lm infection. In preliminary experiments, Btk-deficient NK cells produced less IFN-γ than wt cells upon IL-12 stimulation in vitro (data not shown), also in agreement with a study showing reduced TLR3-mediated IFN-γ production in NK cells in the absence of Btk [39]. Thus, DCs or neutrophils rather than NK cells might be responsible for enhanced serum IFN-γ levels and increased survival in Btk-deficient mice.

Upon infection of macrophages, *Lm* escapes the phagosome and replicates in the cytoplasm of infected cells. Pattern recognition receptors involved in recognition of *Lm* are TLR2 and intracellular receptors such as NOD-like receptors (NLR) and RIG-I-like receptors (RLR) [19], [20], [21]. Macrophages display distinct signaling and transcriptional responses to phagosomal and cytosolic *Lm* [18], including MyD88-dependent pathways originating from the cell surface and the phagosome, the activation of MAPK pathways and the induction of NF-κB. Together, this leads to the expression of pro- and anti-inflammatory cytokines such as TNF-α, IL-12 and IL-10. In addition, IRF-3-dependent pathways are induced by cytosolic *Lm*, which results in the expression of IFN-β [19] (Fig. S2). There was no difference in MAPK pathway activation and IFN-I production between wt and Btk-null BMMs upon *Lm* infection. Moreover, the cytokine response to TLR2 ligands such as hk*L*m and Pam_3_CSK_4_ was normal in the absence of Btk, suggesting a role for Btk in signaling mechanisms that are induced by intracellular *Lm*. Since there is reduced phagosomal escape of *Lm* in Btk-null BMMs, the relatively increased phagosomal bacteria might lead to enhanced inflammatory cytokine expression. Moreover, Btk might regulate signaling pathways and transcription factors induced by phagosomal *Lm* such as NF-κB [19]. The identification of biochemical targets of Btk in macrophages upon *Lm* infection using mass spectroscopy approaches will thus be a topic for future investigations.

It has been shown that Btk (as well as Tec) regulates phagocytosis in RAW 264.7 cells [33]. Our study extends this observation to primary macrophages, since Btk-deficient BMMs displayed a reduced uptake of *E. coli* fragments. However, the uptake of *Lm* was similar in wt and Btk-deficient BMMs, thus excluding the possibility that a different uptake rate of *Lm* is the cause for the altered cytokine response we observed. Interestingly, despite similar numbers of *Lm* entering wt and *Btk* ^−/−^, less bacteria were detected in the cytoplasm of *Btk* ^−/−^ BMMs, indicating reduced phagosomal escape compared to wt cells. It is known that activated BMMs can inhibit the escape of *Lm* via ROS and NO intermediates [40]. However, NO levels were similar in infected wt and *Btk* ^−*/*−^ BMMs. Moreover, the elevated cytokine levels in *Btk* ^−/−^ BMMs appear not to be linked with the impaired phagosomal escape, since wt cells infected in the presence of exogenous TNF-α, IL-6 and IL-12 did not show impaired phagosomal escape (Fig. S3).

Btk has been shown in several cell lineages to have a positive regulatory function in many signaling pathways [1], [2]. Our data demonstrating Btk as a negative regulator of innate immune responses upon *Lm* infection is in line with previous reports showing that Btk displays also negative regulatory roles upon TLR stimulation in human Btk-deficient monocytes [13], [14] or neutrophils [3]. Thus, dependent on the cellular context and the type of activation, Btk can either have a positive or a negative regulatory function. Interestingly, we did not observe activation of Tec, which is the only other member of the Tec kinase family expressed in BMMs [30], upon *Lm* infection (preliminary data not shown). This might suggest a unique function for Btk during *Lm* infection in comparison to other Tec family kinases. Further studies will be important to investigate a potential role for Tec in macrophages during *Lm* infection.

Finally, our observation that Btk-deficient mice display higher protection to *Lm* infection is contradicting an earlier study reporting an enhanced susceptibility of *Btk* ^−*/*−^ mice [41]. Bao et al. characterized a novel PDCA^+^ Siglec-H^–^ B cell subset that produces IFN-α and enhances the innate immune response against *Lm* via IFN-α-mediated activation of NK cells. This B cell subset is reduced in Btk-deficient mice. However, this study did not address the role of macrophages, while our study does not address the role of this B cell subset. The reason for the discrepancies in the *in vivo* phenotypes is not known and might be related to differences in the purity of the genetic C57BL/6 background or the sex of the Btk-null mice (we used male mice for *in vivo* experiments, while the sex has not been specified by Bao et al.), the *Lm* strains (the strain EGD was used for *in vivo* infections in our study, while the *Lm* strain has not been specified in the other study) and burdens (10^6^ in our study versus 2×10^6^ in the other study) used for infection, differences in the microbiota, or to other unknown factors. Btk is expressed in multiple cell lineages of the hematopoietic system and can positively and negatively regulate immune cell function. Thus, conditional gene targeting approaches will be necessary in future experiments to dissect the role of Btk in individual immune cell types following infections.

In summary, our study indicates an important regulatory role for Btk in macrophages in response to *Lm* infection and thus further expands the functional roles of Btk during the regulation of the immune response to microbial infections.

## Supporting Information

Figure S1 **Impaired escape of** ***Lm*** **from phagosomes of** ***Btk^−/−^*** **BMMs.** (A) Wt and *Btk* ^−*/*−^ BMMs were infected with *Lm* (LO28, MOI 40) and three hours later cells were fixed, permeabilized and cellular actin was stained with Phalloidin-Alexa (red). Bacteria were stained using an α-*Lm* antibody and a corresponding secondary antibody (AlexaFluor 488, green). The total number of intracellular bacteria and the number of cytoplasmic bacteria co-localizing with host actin (yellow or surrounded by a red actin cloud) were determined by confocal microscopy as described in material and methods. The diagram on the left indicates the mean number of *Lm* per cell. The right diagram displays the percentage of *Lm* that escaped to the cytoplasm. For each experiment, the number of *Lm* in 50 infected wt or *Btk* ^−*/*−^ BMMs was counted. Data are representative of two independent experiments. (B) Representative confocal microscopical images of the localization of *Lm* in infected (LO28, MOI 40) wt and *Btk* ^−*/*−^ BMMs. Cells were handled as described in (A) and images were obtained at a 40× magnification. Arrows indicate escaped bacteria (yellow or surrounded by a red actin cloud). (A and B) Mean with SEM is shown. The P-values were calculated using an unpaired Student's t-test. ***, P≤0.001; n.s. not significant.
(TIF)

Figure S2 **Schematic drawing showing possible interactions of Btk with** ***Lm*** **-induced innate signaling pathways.** *Lm* induces TLR2 signaling and several intracellular signaling pathways. TLR2 signaling results in MyD88-mediated activation of NF-κB or MAPK pathways, leading to the production of pro-inflammatory cytokines. *Lm* engulfed in the phagosome can also trigger NF-κB signaling. Once bacteria escape from the phagosome to the cytosol, they can be recognized by intracellular pattern recognition receptors such as NOD-like receptors (NLR) or others, leading to IRF-3-induced IFN-β production as well as NF-κB activation. Btk is required for efficient phagosomal escape of *Lm*. Btk might negatively regulate pro-inflammatory cytokine expression via interfering with MyD88-induced or NLR-induced NF-kB activation, although other regulatory modes of Btk are also possible. See manuscript text for details.
(TIF)

Figure S3 **Exogenously added cytokines do not influence** ***Lm*** **uptake or escape.** Wt BMMs were infected with CFSE-labeled *Lm* (LO28, MOI 40) in the presence of TNF-α, IL-6 and IL-12 (the cytokine concentrations were in a range measured in the culture supernatants of Btk-deficient cells; TNF-α 14,7 ng/ml, IL-6 21 ng/ml and IL-12 1,5 ng/ml). Three hours later cells were fixed, permeabilized and cellular actin was stained with Phalloidin-Alexa. The total number of intracellular bacteria and the number of cytoplasmic bacteria co-localizing with host actin were determined as described in material and methods. The diagram on the left indicates the mean number of *Lm* per cell. The right diagram displays the percentage of *Lm* that escaped to the cytoplasm. For each experiment, the number of *Lm* in 50 infected wt or BMMs was counted. Data show summary of two independent experiments (performed with two independent batches of wt BMMs). Mean with SEM is shown. The P-values were calculated using an unpaired Student's t-test. **, P≤0.01; n.s. not significant.
(TIF)
