# Supplementary figures and images for: The Tyrosine Kinase Btk Regulates the Macrophage Response to Listeria monocytogenes Infection
Source: PLoS One. 2013 Mar 27;8(3):e60476. doi: 10.1371/journal.pone.0060476 (PMC3609758; doi:10.1371/journal.pone.0060476)

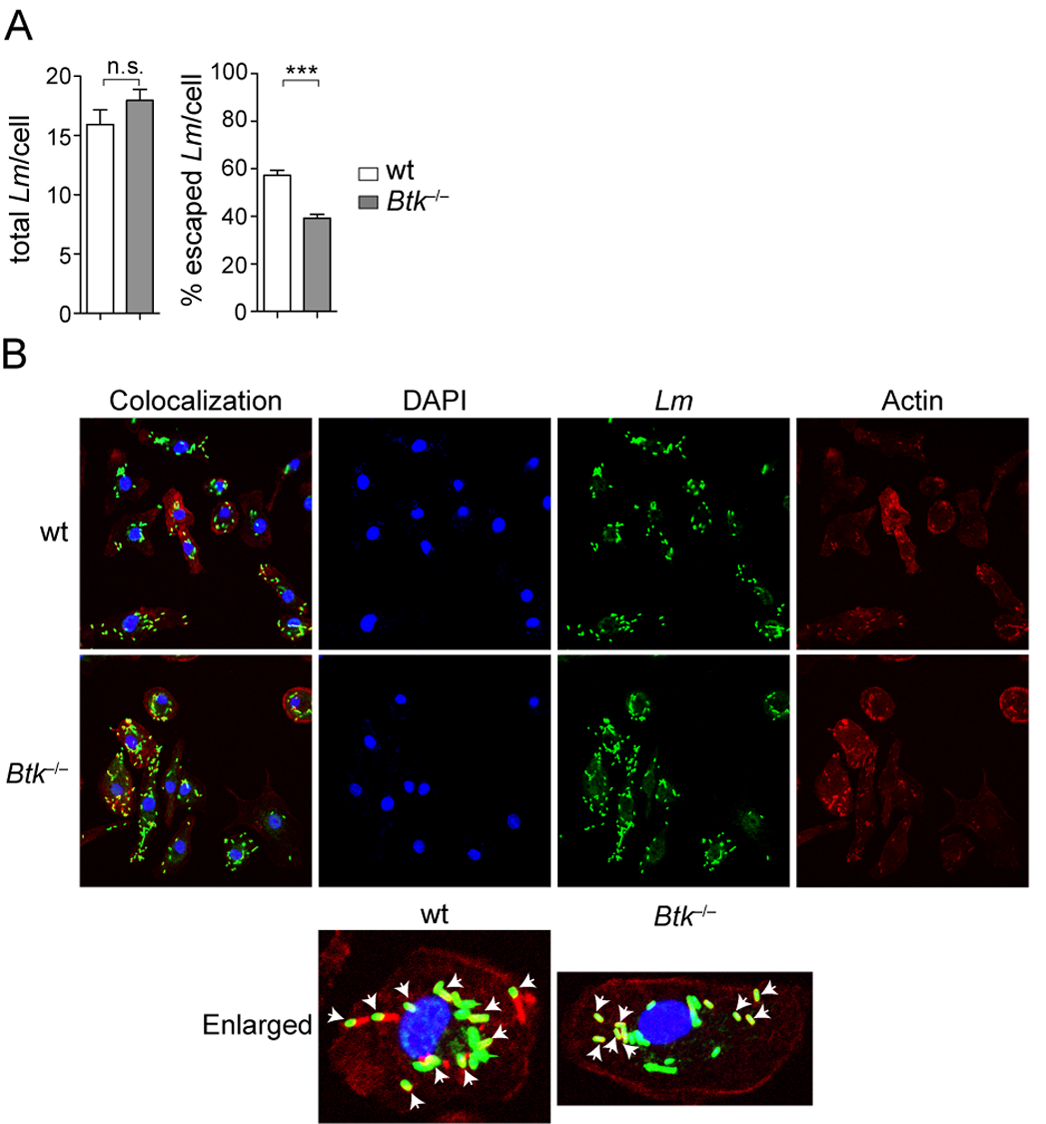

Supplement: Figure S1 — Impaired escape of Lm from phagosomes of Btk−/− BMMs. (A) Wt and Btk −/− BMMs were infected with Lm (LO28, MOI 40) and three hours later cells were fixed, permeabilized and cellular actin was stained with Phalloidin-Alexa (red). Bacteria were stained using an α-Lm antibody and a corresponding secondary antibody (AlexaFluor 488, green). The total number of intracellular bacteria and the number of cytoplasmic bacteria co-localizing with host actin (yellow or surrounded by a red actin cloud) were determined by confocal microscopy as described in material and methods. The diagram on the left indicates the mean number of Lm per cell. The right diagram displays the percentage of Lm that escaped to the cytoplasm. For each experiment, the number of Lm in 50 infected wt or Btk −/− BMMs was counted. Data are representative of two independent experiments. (B) Representative confocal microscopical images of the localization of Lm in infected (LO28, MOI 40) wt and Btk −/− BMMs. Cells were handled as described in (A) and images were obtained at a 40× magnification. Arrows indicate escaped bacteria (yellow or surrounded by a red actin cloud). (A and B) Mean with SEM is shown. The P-values were calculated using an unpaired Student's t-test. ***, P≤0.001; n.s. not significant. (TIF) [file pone.0060476.s001.tif]

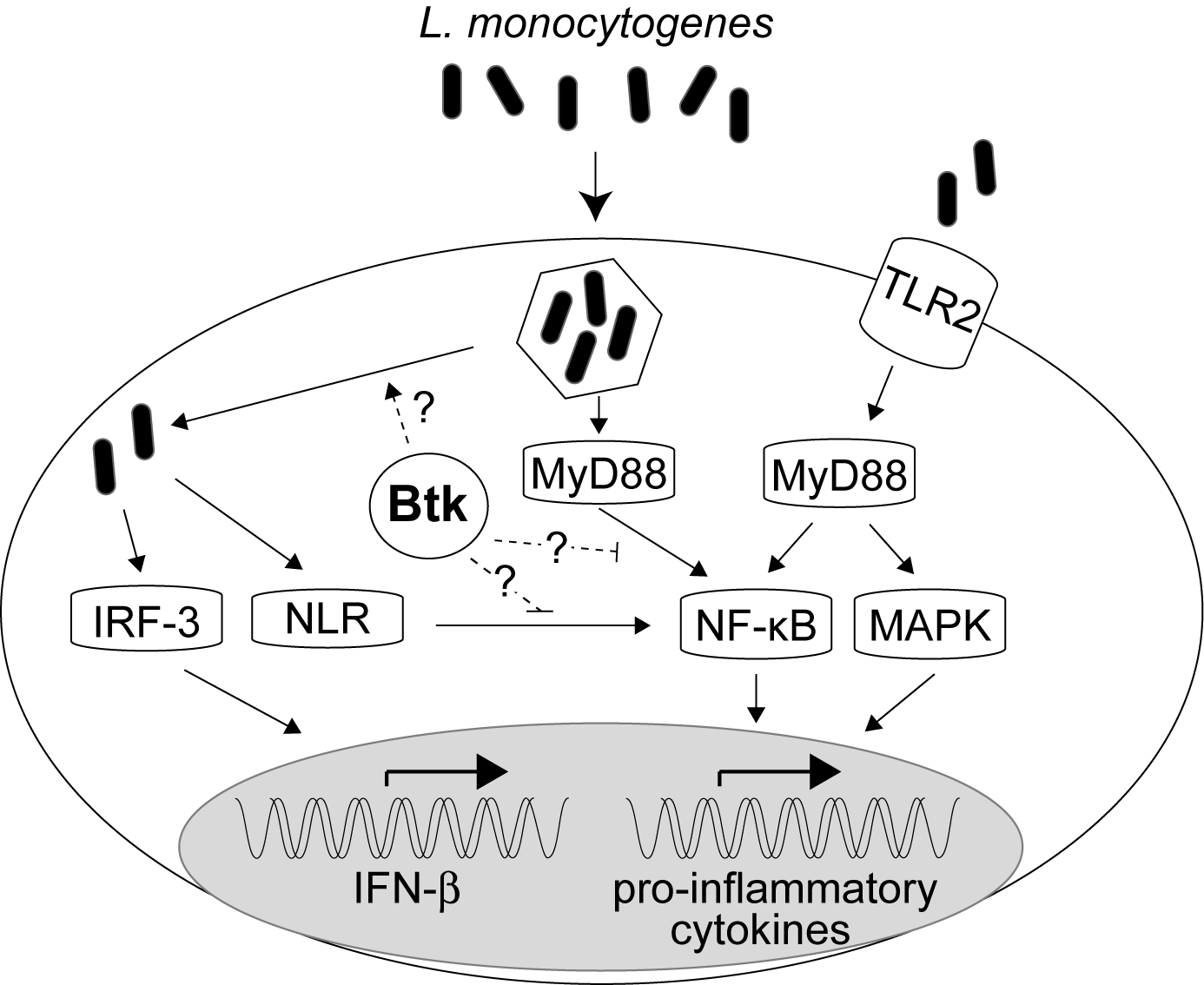

Supplement: Figure S2 — Schematic drawing showing possible interactions of Btk with Lm -induced innate signaling pathways. Lm induces TLR2 signaling and several intracellular signaling pathways. TLR2 signaling results in MyD88-mediated activation of NF-κB or MAPK pathways, leading to the production of pro-inflammatory cytokines. Lm engulfed in the phagosome can also trigger NF-κB signaling. Once bacteria escape from the phagosome to the cytosol, they can be recognized by intracellular pattern recognition receptors such as NOD-like receptors (NLR) or others, leading to IRF-3-induced IFN-β production as well as NF-κB activation. Btk is required for efficient phagosomal escape of Lm. Btk might negatively regulate pro-inflammatory cytokine expression via interfering with MyD88-induced or NLR-induced NF-kB activation, although other regulatory modes of Btk are also possible. See manuscript text for details. (TIF) [file pone.0060476.s002.tif]

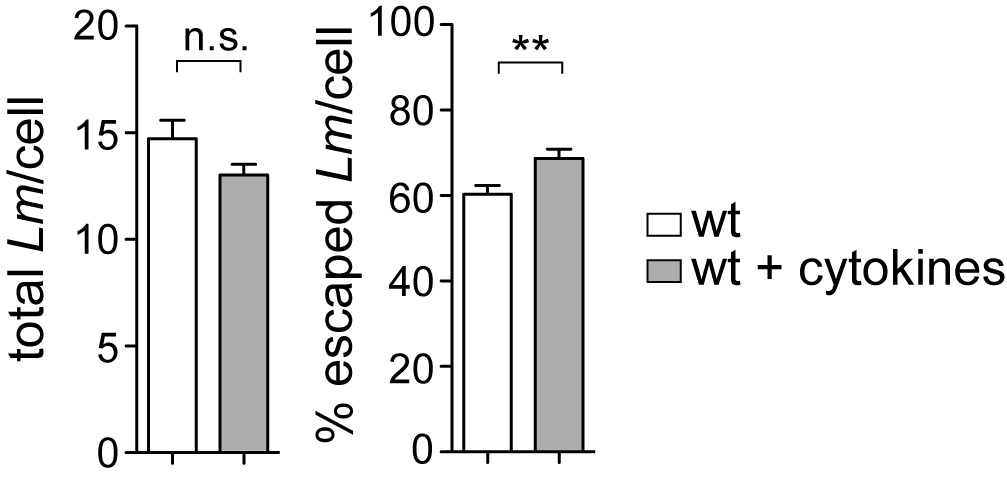

Supplement: Figure S3 — Exogenously added cytokines do not influence Lm uptake or escape. Wt BMMs were infected with CFSE-labeled Lm (LO28, MOI 40) in the presence of TNF-α, IL-6 and IL-12 (the cytokine concentrations were in a range measured in the culture supernatants of Btk-deficient cells; TNF-α 14,7 ng/ml, IL-6 21 ng/ml and IL-12 1,5 ng/ml). Three hours later cells were fixed, permeabilized and cellular actin was stained with Phalloidin-Alexa. The total number of intracellular bacteria and the number of cytoplasmic bacteria co-localizing with host actin were determined as described in material and methods. The diagram on the left indicates the mean number of Lm per cell. The right diagram displays the percentage of Lm that escaped to the cytoplasm. For each experiment, the number of Lm in 50 infected wt or BMMs was counted. Data show summary of two independent experiments (performed with two independent batches of wt BMMs). Mean with SEM is shown. The P-values were calculated using an unpaired Student's t-test. **, P≤0.01; n.s. not significant. (TIF) [file pone.0060476.s003.tif]
